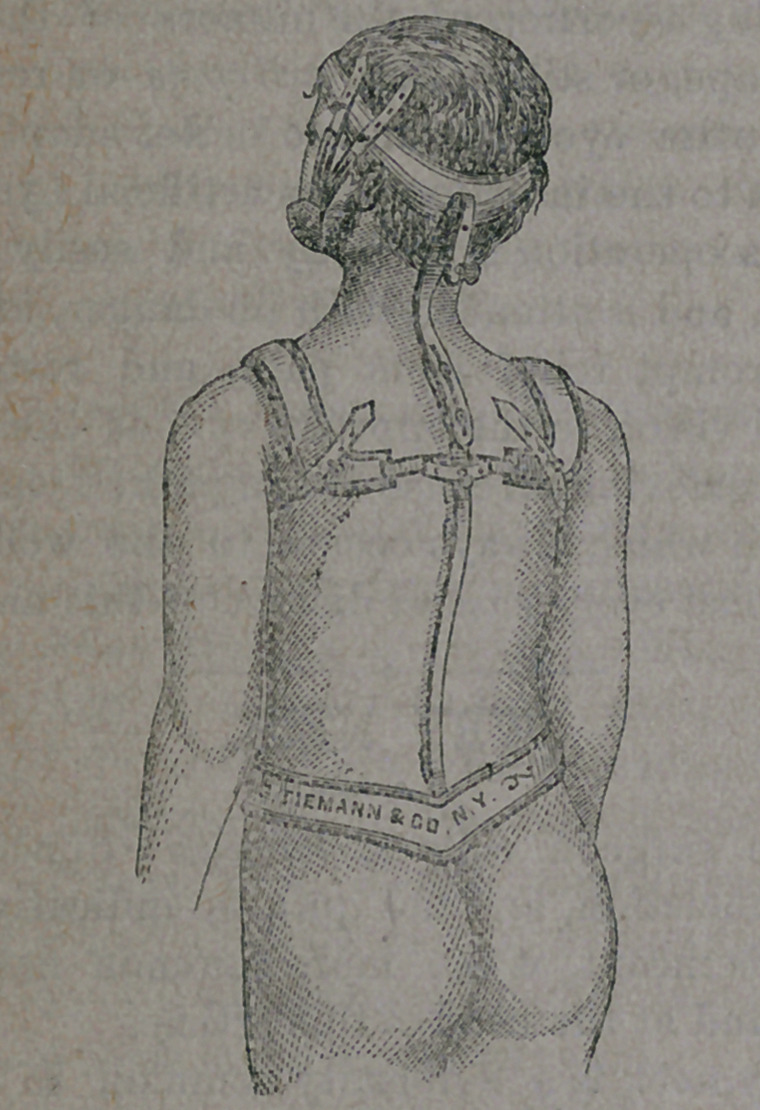# Wry Neck

**Published:** 1874-01

**Authors:** 


### WRY NECK.

The surgeon calls it *torticollis*, difficult of pronunciation, and not quite so indicative of what is meant, as the more common name to be found at the head of this article.

Wry neck is a deformity common to both sexes, but is most prevalent among young girls, children sometimes being born with it. It consists in a contraction of certain muscles of the neck, especially the *sterno-cleido-mastoid,* which drags the head in the direction of the shoulder, at the same time throwing the chin forward in such a manner as to give an extremely disagreeable expression to the face of the person so affected. It may be induced in childhood, by keeping the head constrained in one position, by reason of the presence of a boil, abscess, or other painful sore upon the neck. Rheumatism, neuralgia and paralysis are also fruitful causes for the deformity. It is one very frequently met with, and is so easily remedied, that a simple description of the means employed is all that is necessary, we hope, to induce parents to seek proper means of relief. The patient is placed under the influence of ether or chloroform, the head is then forced in an opposite direction to the contracted muscle, which is by this means, better exposed. A delicate knife is then thrust through the skin, and under the muscle, which is cut in twain from behind forward, allowing the head and neck to assume its natural position. The operation is simple, bloodless, painless, and attended with not the least danger. The muscle being severed under the skin, (subcutaneously) not a drop of blood is seen, and the puncture made, is scarcely observable when completed. By this means the head and neck are made immediately straight, and all trace of deformity at once banished. A suitable apparatus is now adjusted, as here figured :

which keeps the head in its proper position while the healing process is going on. This is worn for a few weeks, or until the gap made in the cut muscle is completely filled, so preventing any liability to a re-shortening and consequent contraction of the faulty muscle.

Why parents allow their children to remain deformed, roaming about our streets, to the horror and often disgust of sensitive people, can only be accounted for upon the hypothesis that they are ignorant of the fact that many, if not all, of these unsightly imperfections of body and limb are amenable to treatment, in the hands of competent surgeons, at this time. We are endeavoring, through the columns of the Bistoury, to familiarize the laity with the vast improvements that have recently been made in the treatment of orthopædic difficulties, in order that the fact may become generally known, that means of cure are at hand, for many of those deformities that heretofore have been pronounced beyond the surgeon's skill.

## Figures and Tables

**Figure f1:**